# Bibliometric analysis of the application of deep learning in cancer from 2015 to 2023

**DOI:** 10.1186/s40644-024-00737-0

**Published:** 2024-07-04

**Authors:** Ruiyu Wang, Shu Huang, Ping Wang, Xiaomin Shi, Shiqi Li, Yusong Ye, Wei Zhang, Lei Shi, Xian Zhou, Xiaowei Tang

**Affiliations:** 1 Department of Gastroenterology, The Affiliated Hospital of Southwest Medical University, Street Taiping No.25, Region Jiangyang, Luzhou, Sichuan Province 646099 China; 2 Nuclear Medicine and Molecular Imaging Key Laboratory of Sichuan Province, Luzhou, China; 3 Department of Gastroenterology, Lianshui County People’ Hospital, Huaian, China; 4 Department of Gastroenterology, Lianshui People’ Hospital of Kangda CollegeAffiliated to, Nanjing Medical University , Huaian, China

**Keywords:** Deep learning, Cancer, Imaging, Bibliometric analysis, VOSviewer, CiteSpace

## Abstract

**Background:**

Recently, the application of deep learning (DL) has made great progress in various fields, especially in cancer research. However, to date, the bibliometric analysis of the application of DL in cancer is scarce. Therefore, this study aimed to explore the research status and hotspots of the application of DL in cancer.

**Methods:**

We retrieved all articles on the application of DL in cancer from the Web of Science database Core Collection database. Biblioshiny, VOSviewer and CiteSpace were used to perform the bibliometric analysis through analyzing the numbers, citations, countries, institutions, authors, journals, references, and keywords.

**Results:**

We found 6,016 original articles on the application of DL in cancer. The number of annual publications and total citations were uptrend in general. China published the greatest number of articles, USA had the highest total citations, and Saudi Arabia had the highest centrality. *Chinese Academy of Sciences* was the most productive institution. *Tian, Jie* published the greatest number of articles, while *He Kaiming* was the most co-cited author. *IEEE Access* was the most popular journal. The analysis of references and keywords showed that DL was mainly used for the prediction, detection, classification and diagnosis of breast cancer, lung cancer, and skin cancer.

**Conclusions:**

Overall, the number of articles on the application of DL in cancer is gradually increasing. In the future, further expanding and improving the application scope and accuracy of DL applications, and integrating DL with protein prediction, genomics and cancer research may be the research trends.

## Introduction

Artificial intelligence (AI), an important branch of computer science, involves algorithms capable of analyzing complex data, which was first introduced by *McCarthy* in 1956 [1]. AI has experienced rapid development from machine learning (ML) to deep learning (DL) [2, 3]. DL, a sub-branch of AI and ML, was first introduced by *Hinton* in 2006 [4]. In 2012, *Krichevsky* designed a new *ImageNet* in the *ILSVRC-2012* competition, and won the first place with an error rate nearly 10% lower than the second place, marking the entry of AI into a new phase of DL [5]. DL forms complex layers through layer-by-layer training, using the upper layer's training result as an initialization parameter for the lower layer training process, thereby obtaining more representative feature data [6–8]. Currently, commonly used algorithms for DL include convolutional neural networks (CNNs) and recurrent neural networks (RNNs) [9]. To date, DL has made outstanding progress in computer vision, speech recognition, natural language processing, and biomedicine [9–12]. In biomedicine, the application of DL spans electronic information file management, medical imaging, disease analysis, genomics, and robotic-assisted surgery [13–15].

Cancer remains a leading cause of death in the world, with its incidence increasing annually and affecting younger populations. The extensive research on DL has led to the wide application of DL in cancer prediction, detection, classification, diagnosis, and prognosis, particularly due to its significant advantages in medical imaging. At present, DL has been applied to various cancers including breast cancer, skin cancer, lung cancer, prostate cancer, cervical cancer, gastric cancer and colorectal cancer [16–20]. However, despite the application of DL in cancer has achieved some achievements, there are still challenges. The application scope, accuracy, and optimal algorithms of DL still need to be further explored and improved [21, 22]. Therefore, it is necessary and important to conduct a bibliometric analysis to summarize the current research status and hotspots, which will further strengthen the research on the application of DL in cancer.

Bibliometric analysis uses mathematical and statistical methods to quantitatively analyze the publications in a field, and can explore research status, hotspots, and trends through co-occurrence analysis, cluster analysis, timeline graph, and burst detection. To our knowledge, bibliometric analysis has been used in various fields, including the application of DL in specific cancer types such as breast cancer, lung cancer, colorectal cancer and gastrointestinal cancer [23–25]. However, a bibliometric analysis on the application of DL across all cancer types at an overall level has not yet been conducted. To address this gap, we planned to perform a bibliometric analysis of the application of DL in all cancer types, followed by specific analyses of individual cancer types. Therefore, in this study, we aimed to explore the research status and hotspots of the application of DL in cancer through bibliometric analysis, providing researchers with landmark articles and key topics, high-impact institutions and influential authors, and even inspire some new inspiration. More importantly, we hoped to reveal which cancer areas have received more attention and which cancer areas have been overlooked at the overall level, so as to provide directions for subsequent research.

## Materials and methods

### Data source and search strategy

**Fig. 1:**
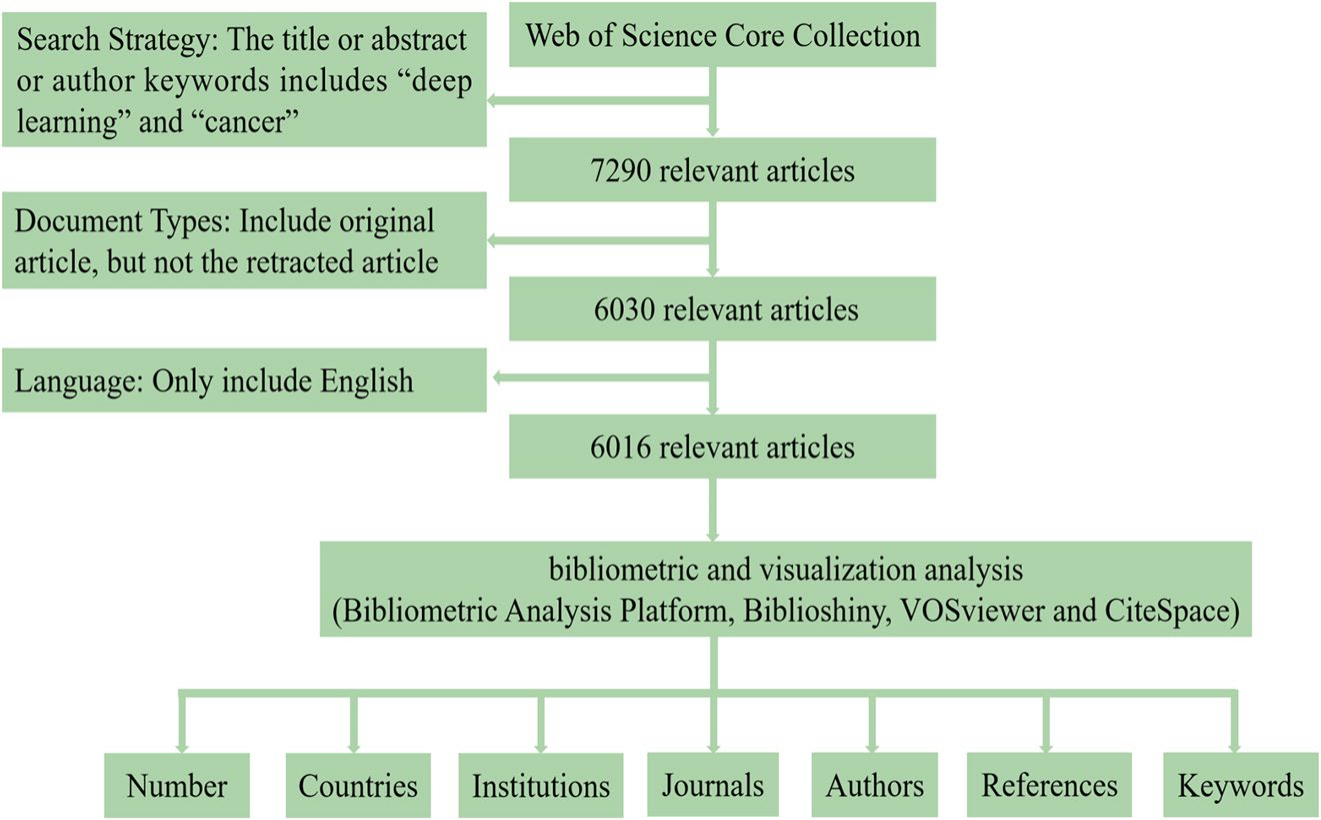
The flowchart of the specific search strategy of the application of deep learning in cancer from the Web of Science Core Collection database

We retrieved the articles on the application of DL in cancer form the Web of Science Core Collection (WoSCC) database. While other databases, such as PubMed, Scopus, and Google Scholar are available, the WoSCC database offers several advantages. The WoSCC database is the largest scientific citation database in the world, known for its extensive coverage of scholarly literature across all disciplines, including biomedical sciences, while covering a variety of journal types such as peer-reviewed journals, conference proceedings, and scholarly publications. The WoSCC database is also recognized for its rigorous selection criteria and quality control measures, ensuring reputable and high-impact article source. In addition, the WoSCC database provides standardized metadata and indexing terminology to facilitate systematic data retrieval and analysis. Therefore, we selected the WoSCC database for our literature search. To avoid the bias due to characteristic of daily updating of the WoSCC database, we completed the literature searches on 16 September 2023. Meanwhile, to comprehensively capture the latest articles and better explore the research trends and hotspots, we included some articles with a publication date later than the search date, mainly including some early access. The specific search strategy was detailed in Fig. 1: First, the title, abstract, or author keywords included “deep learning” and “cancer”. Then, without limiting the timespan, we retrieved all the articles up to 16 September 2023. The document type included only original articles, excluding retracted articles. Only articles in English were included. Two researchers independently examined the title, abstract, and author keywords of each article to determine its relevance to DL and cancer, excluding non-medical uses or irrelevant articles. In cases of disagreement between the two researchers, a third person made the final decision. Finally, we found 6,016 relevant original articles from the WoSCC database, and no duplicate articles were found by the deduplication from CiteSpace.

## Bibliometric and visualization analysis

We exported all records of the above 6016 related articles in plain text files and Excel forms for bibliometric and visualization analysis. We extracted information such as numbers, citations, countries, institutions, authors, journals, references, and keywords. In our study, the analysis of numbers, citations, countries, institutions, authors, journals, and references was based on the extracted raw data. For the analysis of keywords, we merged the original keywords with their respective synonyms to ensure consistency. Then, we conducted bibliometric analysis using the Bibliometric Analysis Platform (bibliometric.com), Biblioshiny, VOSviewer (VOSviewer_1.6.18) and CiteSpace (CiteSpace_6.2. R4). During the analysis, the column and line charts were used to analyze the trends of annual publications and total citations. The Bibliometric Analysis Platform and Biblioshiny mapped publication output in two different forms. VOSviewer and CiteSpace, the two main software that used for bibliometric analysis, were used to conduct co-occurrence analysis, cluster analysis, co-cited analysis, timeline graph, and burst detection. In visualization maps, each node represents a country, institution, author, journal, reference, or keyword. The lines between nodes represent cooperative relationships between the elements, the thicker the line, the closer the cooperative relationships between them. The purple ring on the outside of the node represents centrality, and centrality above 0.1 is considered as high centrality, indicating the central node and high influential. In addition, we obtained the IF 2022 and JCR division data form the Journal Citation Reports.

## Results

### The general global trends

**Fig. 2:**
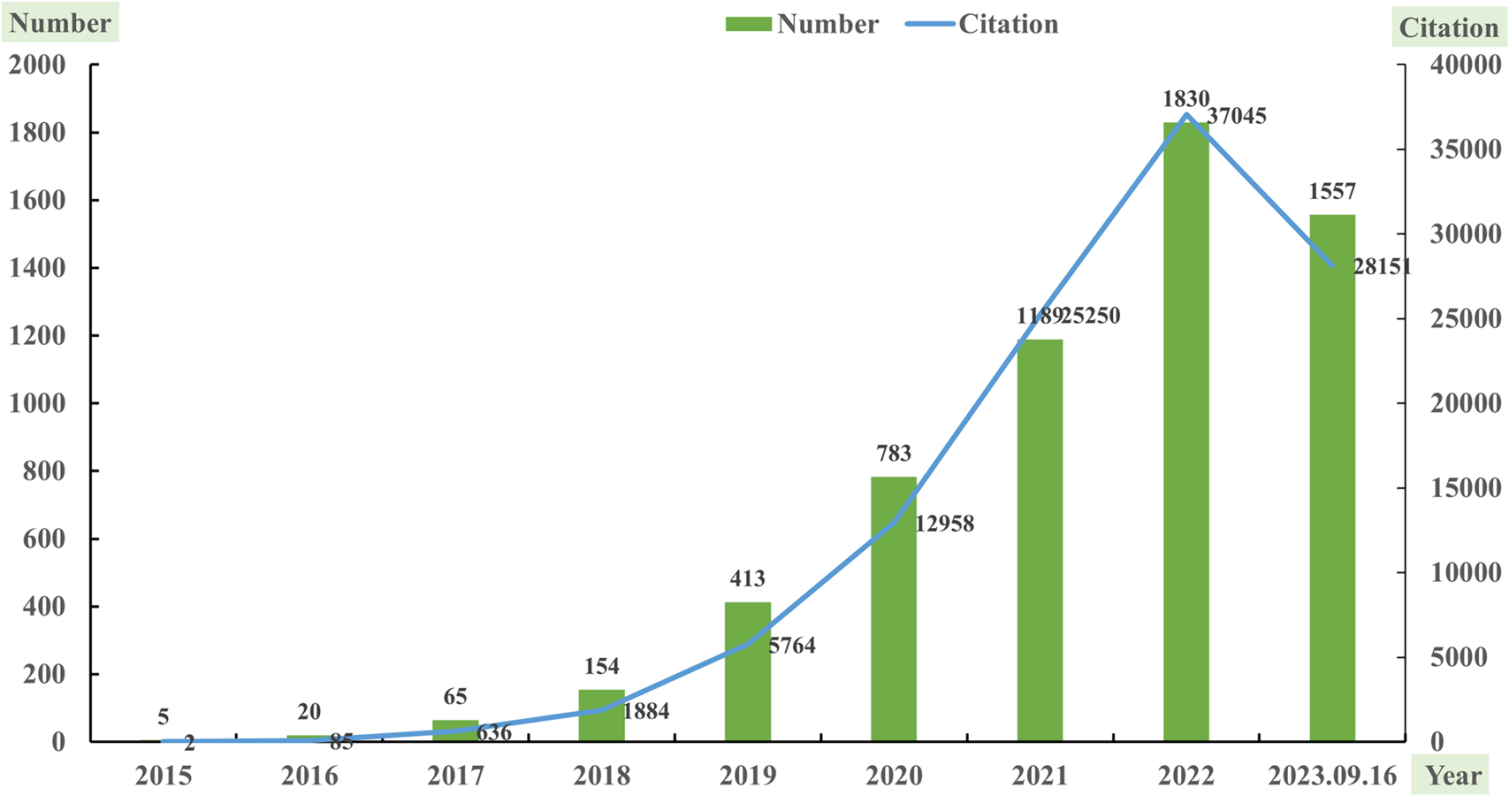
The annual change trends in the number of articles and total citations of application of deep learning in cancer from 2015 to 2023

The number of publications and total citations reflect the development and growing interest in the application of DL in cancer research. We found a total of 6,016 original articles on the application of DL in cancer. From 2015 to 2023, there was a rapid growth in both the number of publications and total citations. As shown in Fig. 2, the number of articles increased from 5 in 2015 to 1,830 in 2022, while total citations increased from 2 in 2015 to 37,045 in 2022, indicating a significant increasing interest in the application of DL in cancer globally. The development stage was roughly divided into three stages: early stage (2015), growth stage (2016–2021), and prosperity stage (2022–2023). There were 1,557 articles and 28,151 total citations in the first nine months of 2023. Based on this data, we forecasted that the total number of articles and total citations in 2023 will continue to grow and eventually surpass those in 2022.

## Analysis of countries

**Fig. 3:**
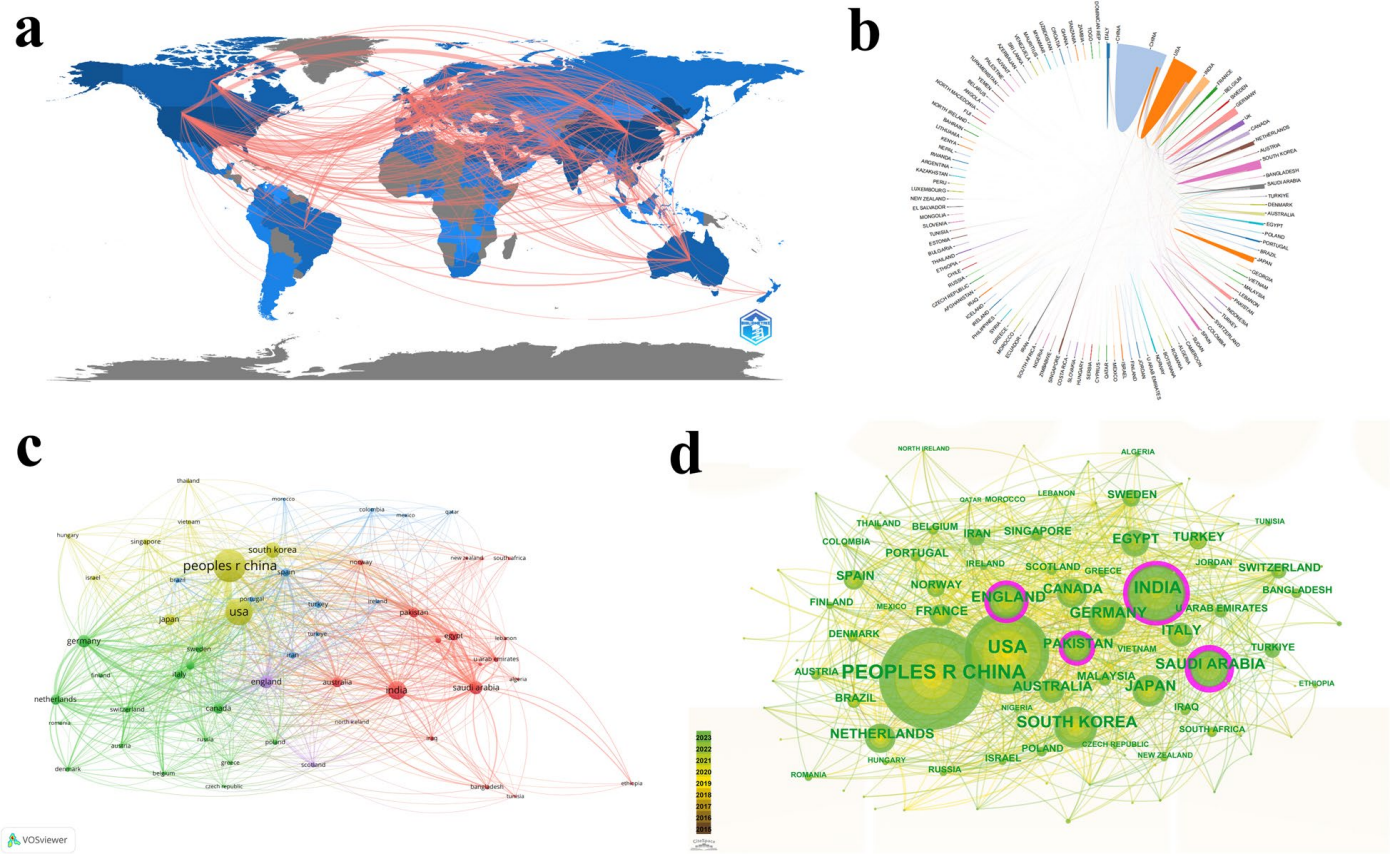
The visualization analysis of countries. **a** The map of country distribution. **b** The cooperation relationships map of countries. **c** The network visualization map of countries. **d** The visualization map of countries. Each node represents a country/region

112 countries/regions published relevant articles on the application of DL in cancer. Table 1 listed the top ten countries by publication volume. More than half of these articles published by China and the USA (57.83%). China was the most productive country with 2,066 articles, followed by USA (1,413), India (676), South Korea (442), and Saudi Arabia (346). USA had the highest total citations (46,820), followed by China (31,072), Netherlands (12,684), England (10,992) and Germany (8,639). Next, we conducted a visualization analysis of the countries. Figure 3a showed the countries/regions that have published articles in blue, and indicated the number of articles by the depth of blue. The pink lines represent the cooperative relationships between countries. Figure 3b also showed the cooperation of countries, with different colored areas representing different countries, region sizes representing the number of articles, and lines representing cooperative relationships. Figure 3c and d was formed using VOSviewer and CiteSpace, respectively. In Fig. 3c, 58 countries/regions were divided into five clusters when the minimum number of a document of a country was ten. In Fig. 3d, Saudi Arabia (0.22), England (0.16), India (0.13) and Pakistan (0.13) showed high centrality, indicating that these countries played more significant roles in the development of the application of DL in cancer. However, although Pakistan had higher centrality, neither its publication volume (194) nor total citations (3,683) were in the top ten.

**Table 1:** The top ten countries by volume of the application of deep learning in cancer

Rank	Country	Number	Total Citations	Mean Citations	Centrality	Total link strength
1	China	2066	31,072	15.04	0.01	936
2	USA	1413	46,820	33.14	0.1	1365
3	India	676	6835	10.11	0.13	446
4	South Korea	442	7809	17.67	0.07	409
5	Saudi Arabia	346	3630	10.49	0.22	579
6	England	336	10,992	32.71	0.16	653
7	Germany	297	8639	29.09	0.07	515
8	Japan	250	4610	18.44	0.02	176
9	Canada	215	4899	22.79	0.07	312
10	Netherlands	206	12,684	61.57	0.04	402

## Analysis of institutions

**Fig. 4:**
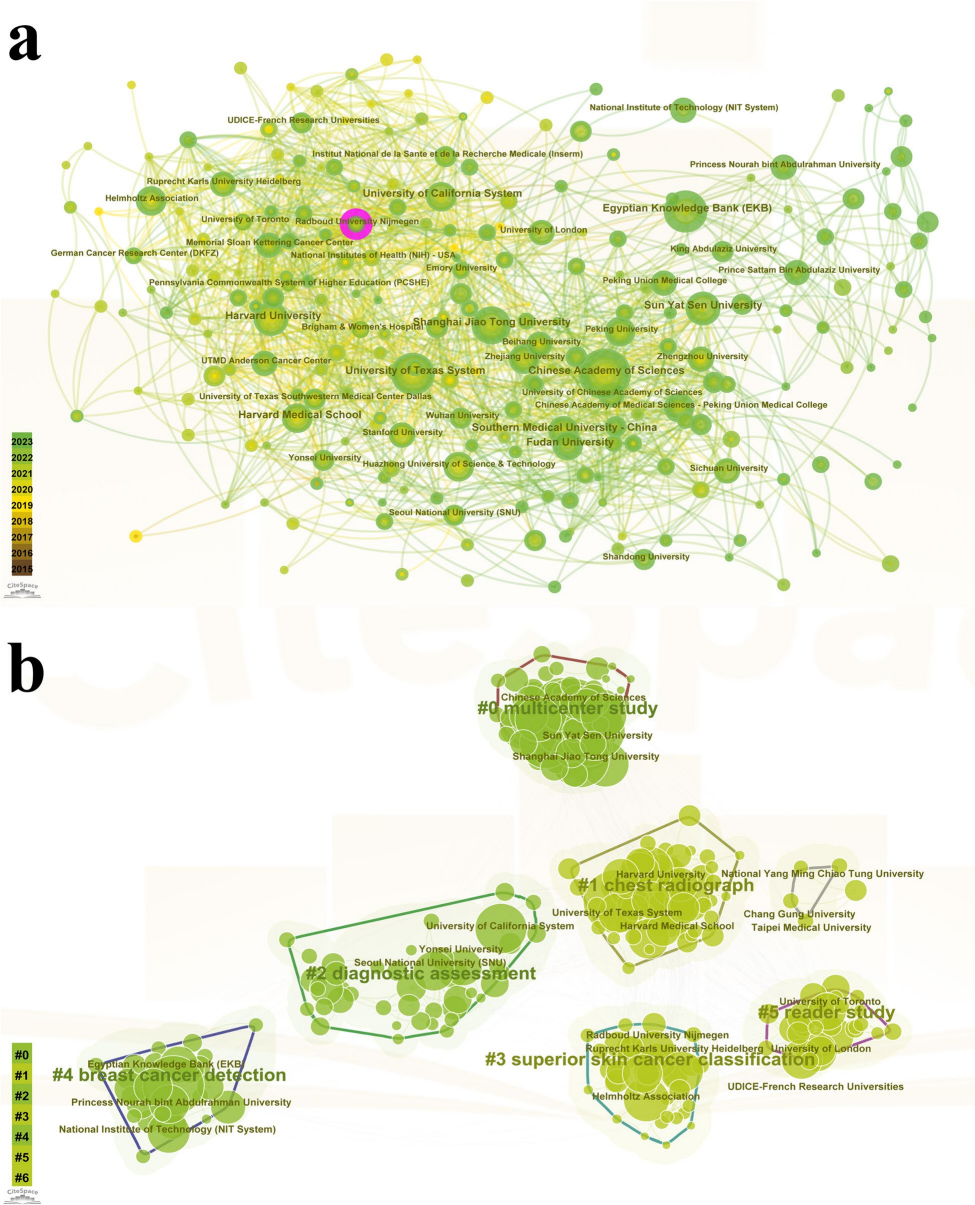
The visualization analysis of institutions. **a** The cooperation relationships network of institutions. **b** The cluster analysis of institutions

6,417 institutions published relevant articles on the application of DL in cancer. Table 2 listed the top ten institutions by publication volume. *Chinese Academy of Sciences* published the greatest number of articles (207), followed by *University of Texas System* (174), *Egyptian Knowledge Bank* (151), *Sun Yat Sen University* (144) and *Shanghai Jiao Tong University* (135). Interestingly, the top ten institutions only affiliated three countries, with five from China, four from USA, and one from Egypt. Subsequently, the visualization analysis of institutions was performed. The active cooperation relationships between the different institutions were shown in Fig. 4a. However, only *Radboud University Nijmegen* (centrality = 0.1, from Netherlands) exhibited high centrality, none of the top ten institutions mentioned above showed high centrality. Figure 4b showed the cluster analysis of institutions and the cluster was significant and convincing (Q = 0.53, S = 0.86. It was generally thought that Q > 0.3 meant the cluster structure was significant, S > 0.5 meant clustering was reasonable, and S > 0.7 was convincing.). The seven clusters were #0 multicenter study; #1 chest radiograph; #2 diagnostic assessment; #3 superior skin cancer classification; #4 breast cancer detection; #5 reader study; #6 did not display an obvious cluster label.

**Table 2:** The top ten institutions and authors by volume of the application of deep learning in cancer

Rank	Institution	Number	Centrality	Country	Author	Number	Total citations	Mean Citations
1	*Chinese Academy of Sciences*	207	0.02	China	*Tian, Jie*	39	1007	25.82
2	*University of Texas System*	174	0.06	USA	*Wang, Jing*	27	273	10.11
3	*Egyptian Knowledge Bank*	151	0.06	Egypt	*Wang, Wei*	27	360	13.33
4	*Sun Yat Sen University*	144	0.06	China	*Lei, Yang*	26	655	25.19
5	*Shanghai Jiao Tong University*	135	0.09	China	*Liu, Tian*	26	648	24.92
6	*Harvard University*	127	0.05	USA	*Yang, Xiaofeng*	25	648	25.92
7	*University of California System*	107	0.03	USA	*Wang, Tonghe*	24	644	26.83
8	*Fudan University*	102	0.02	China	*Khan, Muhammad Attique*	23	900	39.13
9	*Southern Medical University*	98	0.04	China	*Zhang, Yu-dong*	23	810	35.22
10	*Harvard Medical School*	94	0.04	USA	*Liu, Zaiyi*	22	328	14.91

## Analysis of authors

**Fig. 5:**
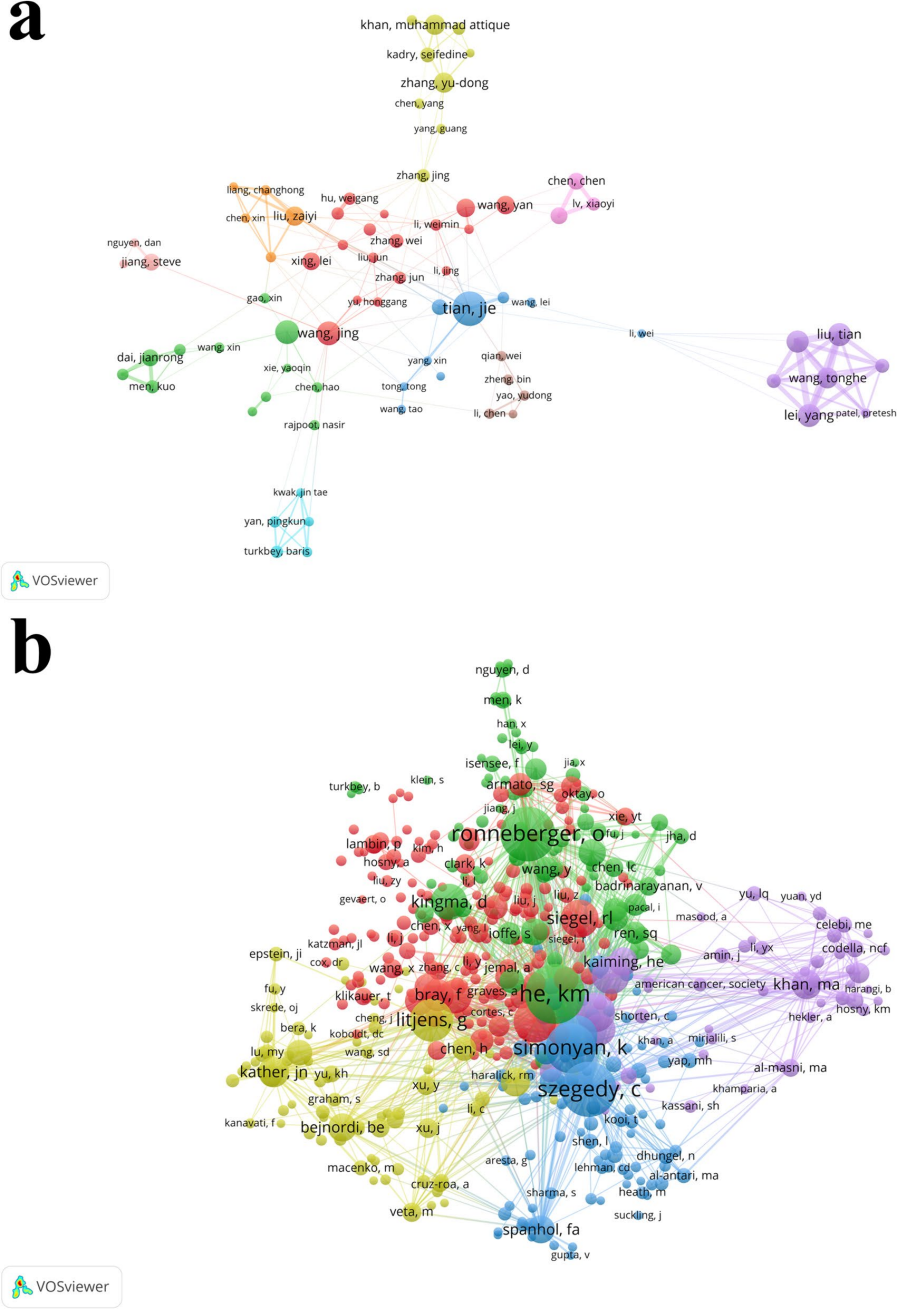
The visualization analysis of authors and co-cited authors. **a** The visualization analysis of authors. **b** The visualization analysis of co-cited authors

28,979 authors contributed to these 6,016 articles. The top ten authors by publication volume were listed in Table 2. *Tian, Jie* published the greatest number of articles (39), followed by *Wang, Jing* (27), *Wang, Wei* (27), *Lei, Yang* (26) and *Liu, Tian* (26). *Tian, Jie* also had the highest total citations (1,008), but *Khan, Muhammad Attique* had the highest mean citations (39.13). Figure 5a showed the visualization analysis of authors. 75 authors were divided into ten clusters when the minimum number of a document of an author was ten. But in fact, 108 authors published more than ten articles. This discrepancy was because some authors were not connected to each other, indicating a relative lack of cooperation relationships between authors. In addition, the co-citation of authors was also analyzed in Fig. 5b. The co-citation meant the authors, journals, or references of two or more articles were cited by another article at the same time. 499 co-cited authors were divided into five cluster when the minimum of citations of an author was fifty, and *He Kaiming* ranked first with 1,201 times co-citations.

## Analysis of journals

**Fig. 6:**
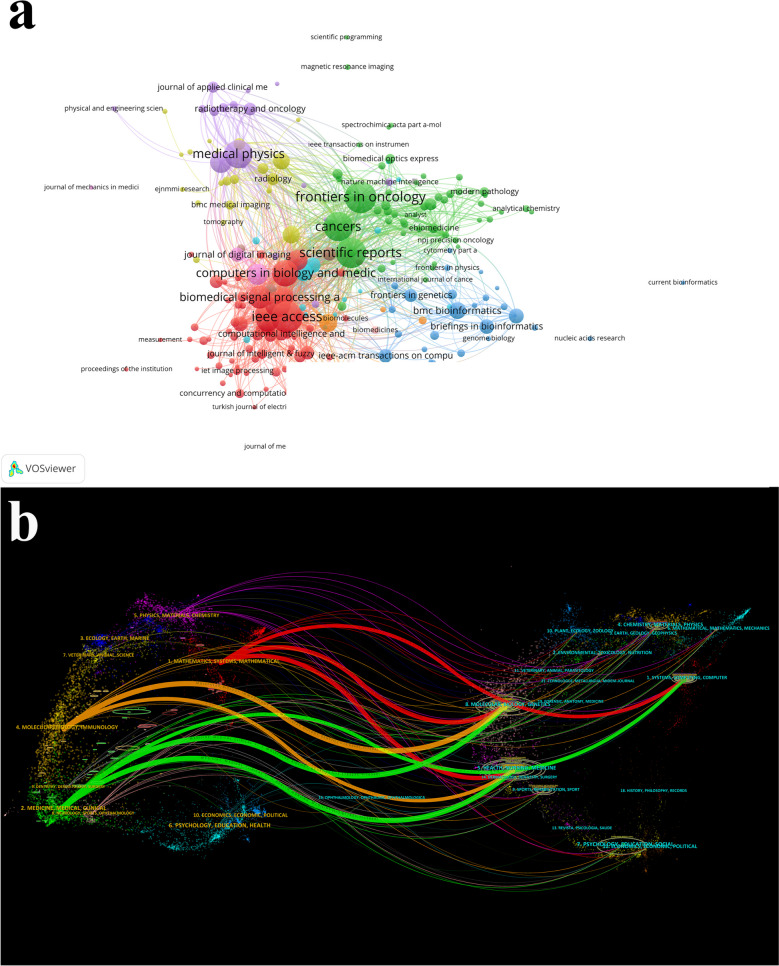
The visualization analysis of journals. **a** The visualization analysis of journals. **b** The analysis of the dual-map overlay of journals

These 6,016 articles were distributed across 821 journals. Table 3 listed the top ten journals by publication volume. *IEEE Access* ranked first with 228 articles, followed by *Frontiers in Oncology* (224), *Scientific Reports* (210), *Cancers* (195), and *Medical Physics* (178). The top ten journals were all classified in JCR Q1 or Q2, and *Computers in Biology and Medicine* had the highest IF 2022(7.7). These journals included not only medical, but also included physics, computer science, and biomedical imaging. The visualization analysis of journals was performed. Figure 6a showed that 220 journals were divided into ten clusters when the minimum of documents of a journal was five. Figure 6b showed the dual-map overlay of journals generated by Citespace, providing a comprehensive view of the temporal and interdisciplinary evolution of the applications of DL in cancer, underscoring the dynamic and interconnected nature of this field. This visualization depicted two distinct datasets, with the left side representing earlier research publications and the right side corresponding to more recent publications. The left side captured the initial integration of DL across various subject areas, reflecting early theoretical foundations, clinical applications, and molecular research that laid the foundation for subsequent advances, such as “Mathematics, Systems, Mathematical” (Cluster 1), “Medicine, Medical, Clinical” (Cluster 2), and “Molecular Biology, Immunology” (Cluster 4). On the right side, it illustrated the evolution and current state of the applications of DL in cancer. Significant clusters included “Systems, Computing, Computer” (Cluster 1), highlighting advanced computational techniques and system-level analyses; “Health, Nursing, Medicine” (Cluster 5), focusing on the clinical applications and healthcare implications of DL; and “Molecular Biology, Genetics” (Cluster 8), delving into genetic research and its integration with DL technologies.

**Table 3:** The top ten journals by volume of the application of deep learning in cancer

Rank	Journal	Number	Total citations	IF 2022	JCR division
1	*IEEE Access*	228	3087	3.9	Q2
2	*Frontiers in Oncology*	224	1264	4.7	Q2
3	*Scientific Reports*	210	7281	4.6	Q2
4	*Cancers*	195	1270	5.2	Q1
5	*Medical Physics*	178	4926	3.8	Q2
6	*Computers in Biology and Medicine*	155	2766	7.7	Q1
7	*Diagnostics*	151	899	3.6	Q2
8	*Biomedical Signal Processing and Control*	118	675	5.1	Q2
9	*Multimedia Tools and Applications*	116	972	3.6	Q2
10	*Physics in Medicine and Biology*	106	1902	3.5	Q2

## Analysis of references

**Fig. 7:**
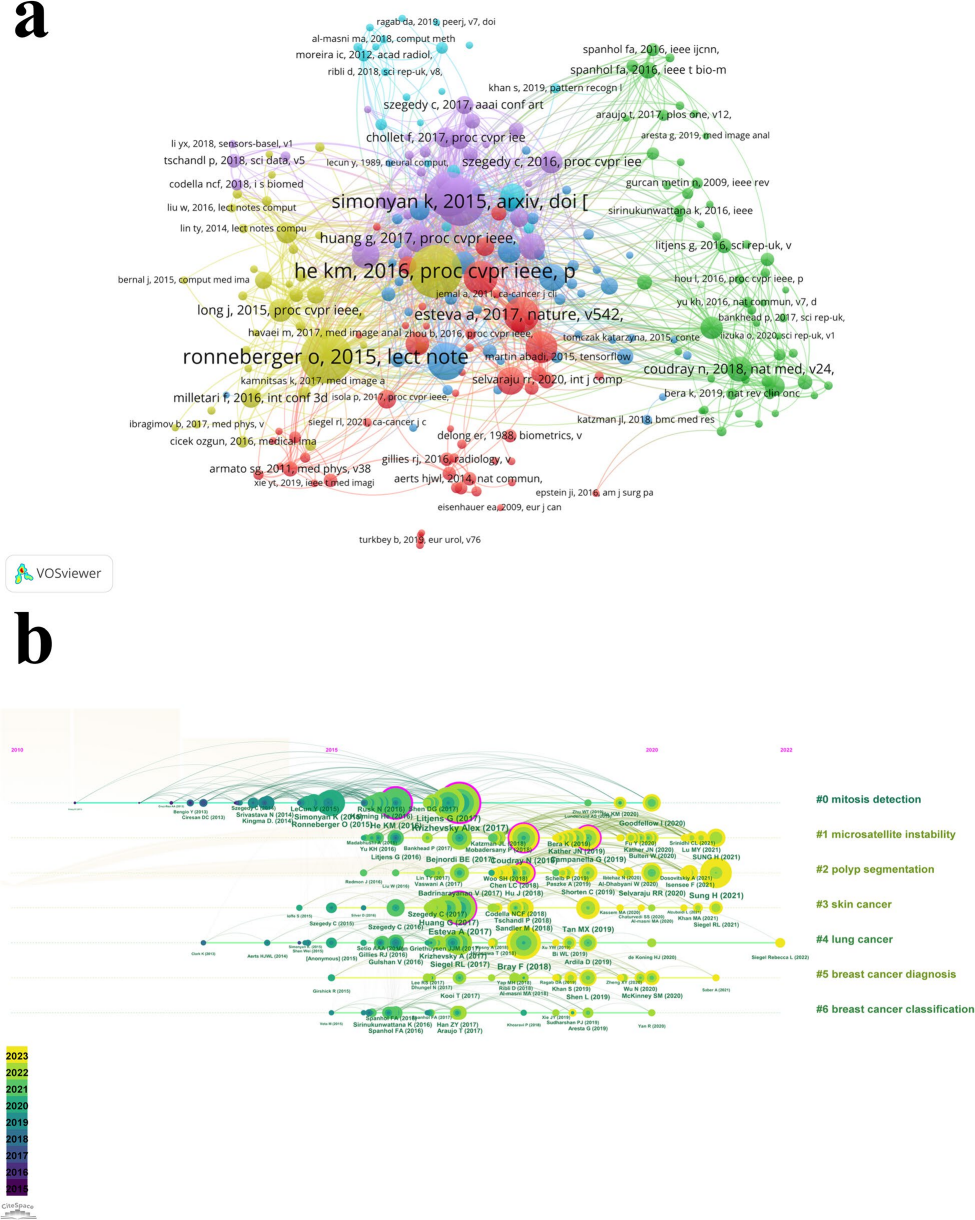
The visualization analysis of references. **a** The visualization analysis of co-cited of references. **b** The timeline graph of references

The times of citations can reflect the influence of an article, the more citations, the more important it was considered. Table 4 listed the top ten most cited articles. The most cited article was “*Computational Radiomics System to Decode the Radiographic Phenotype*” published in 2017 by *Griethuysen*, which has been cited 2,570 times. In addition, the co-citation of references was also an important indicator to reflect the impact of an article and the basis of the research. Table 5 listed the top ten co-cited references. The most co-cited reference was “*ImageNet Classification with Deep Convolutional Neural Network*” published in 2017 by *Krizhevsky*, with 565 co-citations. Interesting, the article published by *Coudray* entitled “*Classification and mutation prediction from non-small cell lung cancer histopathology images using deep learning*” was both one of the top ten cited article and one of the top ten co-cited reference. Then, the visualization map of co-citation of references was shown in Fig. 7a, where the references were divided into six clusters. The timeline graph of references was in Fig. 7b, which clustered references and spread them out in chronological order, showing the change of cluster over time. From Fig. 7b, we can identify the most co-cited references (with larger nodes), references with high centrality (purple rings on the outside of the node), and the clustering label. The seven clusters were: #0 mitosis detection; #1 microsatellite instability; #2 polyp segmentation; #3 skin cancer; #4 lung cancer; #5 breast cancer diagnosis; #6 breast cancer classification.

**Table 4:** The top ten most cited articles of the application of deep learning in cancer

Rank	Article title	First author	Journal	Count	Year
1	Computational Radiomics System to Decode the Radiographic Phenotype	*Joost JM van Griethuysen*	*Cancer Research*	2570	2017
2	Diagnostic Assessment of Deep Learning Algorithms for Detection of Lymph Node Metastases in Women with Breast Cancer	*Babak Ehteshami Bejnordi*	*Jama-Journal of the American Medical Association*	1402	2017
3	Classification and mutation prediction from non-small cell lung cancer histopathology images using deep learning	*Nicolas Coudray*	*Nature Medicine*	1176	2018
4	Survey on deep learning with class imbalance	*Justin M. Johnson*	*Journal of Big Data*	845	2019
5	Clinical-grade computational pathology using weakly supervised deep learning on whole slide images	*Gabriele Campanella*	*Nature Medicine*	843	2019
6	End-to-end lung cancer screening with three-dimensional deep learning on low-dose chest computed tomography	*Diego Ardila*	*Nature Medicine*	781	2019
7	Pulmonary Nodule Detection in CT Images: False Positive Reduction Using Multi-View Convolutional Networks	*Arnaud A. A. Setio*	*IEEE Transactions on Medical Imaging*	730	2016
8	Deep Patient: An Unsupervised Representation to Predict the Future of Patients from the Electronic Health Records	*Riccardo Miotto*	*Scientific Reports*	723	2016
9	Locality Sensitive Deep Learning for Detection and Classification of Nuclei in Routine Colon Cancer Histology Images	*Korsuk Sirinukunwattana*	*IEEE Transactions on Medical Imaging*	692	2016
10	Artificial intelligence in cancer imaging: Clinical challenges and applications	*Wenya Linda Bi*	*Ca-a Cancer Journal for Clinicians*	575	2019

**Table 5:** The top ten co-cited references of the application of deep learning in cancer

Rank	Article title	First author	Journal	Count	Year
1	ImageNet Classification with Deep Convolutional Neural Networks	*Alex Krizhevsky*	*Communications of the ACM*	565	2017
2	Dermatologist-level classification of skin cancer with deep neural networks	*Andre Esteva*	*Nature*	403	2017
3	Global Cancer Statistics 2018: GLOBOCAN Estimates of Incidence and Mortality Worldwide for 36 Cancers in 185 Countries	*Freddie Bray*	*Ca-a Cancer Journal for Clinicians*	403	2018
4	A survey on deep learning in medical image analysis	*Geert Litjens*	*Medical Image Analysis*	368	2017
5	Global Cancer Statistics 2020: GLOBOCAN Estimates of Incidence and Mortality Worldwide for 36 Cancers in 185 Countries	*Hyuna Sung*	*Ca-a Cancer Journal for Clinicians*	334	2021
6	Deep Residual Learning for Image Recognition	*Kaiming He*	*2016 IEEE Conference on Computer Vision and Pattern Recognition*	309	2016
7	Classification and mutation prediction from non–small cell lung cancer histopathology images using deep learning	*Nicolas Coudray*	*Nature Medicine*	298	2018
8	Densely Connected Convolutional Networks	*Gao Huang*	*2017 IEEE Conference on Computer Vision and Pattern Recognition*	268	2016
9	U-Net: Convolutional Networks for Biomedical Image Segmentation	*Olaf Ronneberger*	*Lecture Notes in Computer Science*	244	2015
10	Very deep convolutional networks for Large-Scale image recognition	*Karen Simonyan*	*Computer Vision and Pattern Recognition*	220	2015

## Analysis of keywords

**Fig. 8:**
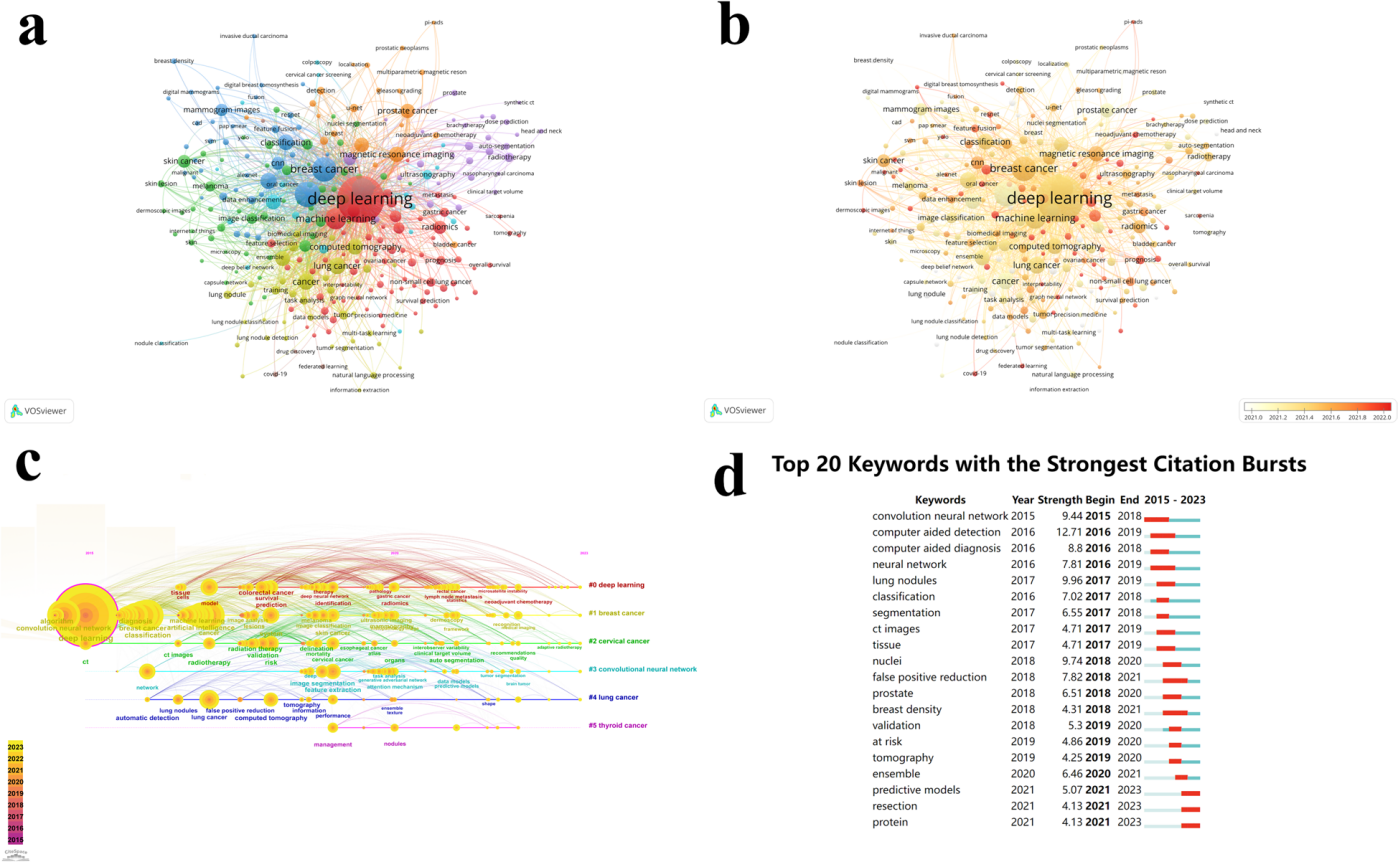
The visualization analysis of keywords. **a** The network visualization map of keywords. **b** The overlay visualization map of keywords. **c** The timeline graph of keywords. (Dd) The top twenty keywords with the strongest citation bursts

Keywords can directly express the topic of an article, and analysis of keywords can reveal the research hotspots and trends in a certain field. After extracting and merging the keywords, the top thirty keywords with the most frequency were listed in Table 6. “Deep learning” was the most frequent keyword, followed by “convolution neural network”, “breast cancer”, “artificial intelligence”, “machine learning”, “lung cancer”, “cancer”, “transfer learning”, “computed tomography”, “classification”. Among these keywords, six keywords were directly related to cancer: “breast cancer”, “lung cancer”, “prostate cancer”, “skin cancer”, “cervical cancer”, and “colorectal cancer”. Then, the visualization analysis was performed using VOSviewer. The network visualization map (Fig. 8a) showed 276 keywords were divided into nine clusters when the minimum number of occurrences of a keyword was ten. The red cluster including “deep learning” was the largest cluster, with 78 keywords. The overlay visualization map (Fig. 8b) added the time factor into the analysis, with lighter colors indicating earlier occurrences and redder colors indicating later occurrences. It is evident that DL has been studied earlier in breast cancer, skin cancer, and lung cancer, while colorectal cancer and oral cancer have been studied relatively late. The visualization analysis was also performed using CiteSpace. The timeline graph (Fig. 8c) clustered keywords and expanded them in chronological order to show the change of hotspots and the process of development. The six clusters were #0 deep learning; #1 breast cancer; #2 cervical cancer; #3 convolutional neural network; #4 lung cancer; #5 thyroid cancer. The burst detection was shown in Fig. 8d, where listed the top twenty keywords with the strongest citation bursts. “Computer aided detection” was the keyword with the strongest citation bursts appearing in 2015. “Predictive models”, “resection”, and “protein” were among the recent keywords with stronger citation bursts.

**Table 6:** The top thirty keywords with the most frequency of the application of deep learning in cancer

Rank	Keywords	Count	Rank	Keywords	Count	Rank	Keywords	Count
1	deep learning	3181	11	magnetic resonance imaging	240	21	image segmentation	126
2	convolution neural network	908	12	computer-aided diagnosis	215	22	colorectal cancer	125
3	breast cancer	682	13	feature extraction	212	23	digital pathology	110
4	artificial intelligence	535	14	segmentation	209	24	image classification	106
5	machine learning	485	15	prostate cancer	199	25	histopathological image	101
6	lung cancer	289	16	radiomics	164	26	melanoma	90
7	cancer	271	17	skin cancer	156	27	deep neural network	88
8	transfer learning	266	18	mammogram images	140	28	whole-slide image	87
9	computed tomography	250	19	cervical cancer	137	29	medical image	84
10	classification	247	20	neural network	135	30	ultrasonography	78

## Discussion

Recently, DL has attracted much attention from the academic community. By building different various models and algorithms, DL has been applied to the prediction, detection, classification, diagnosis, prognosis of cancer, and the discovery of cancer biomarkers, achieving accuracy comparable or even higher than that of clinicians [26, 27]. The application of DL in cancer has not only continuously improved medical diagnosis and medical quality, but also promoted the development of precision medicine [28]. However, with the increasing application of DL in cancer, it is important to understand its current research status and identify the emerging research hotspots. Therefore, we conducted a bibliometric analysis to provide researchers with introductory guidance, an overview of the research status and hotspots and new inspiration of the application of DL in cancer.

Overall, from 2015 to 2023, the number of publications on the application of DL in cancer has increased year by year, and in 2022 it entered a prosperity stage. China, USA, and India are the top three countries that have published articles on the application of DL in cancer, meanwhile China and USA are also the main sources of the top ten institutions, indicating their significant contribution to this field. As one of the most developed country in the world, USA has shown unique advantages in many fields, the rapid development of China in recent years is also well recognized. At the same time, both China and USA proposed relevant strategies to strengthen the development of AI, so it is not surprising that China and USA have performed well in the application of DL in cancer. But why is India in third place? This can be attributed to the high priority of Indian government on AI. The Indian government released “*Digital India Strategy*” in 2015 and “*National Strategy for Artificial Intelligence*” in 2018. In addition to drawing up a blueprint at strategic level, the Indian government has invested heavily in supporting the development of AI [29–32]. This series of measures has enabled India to develop rapidly in AI, including DL. Therefore, to further promote the application of DL in cancer, the support of government policy and financial are very important.

In addition, the cooperation relationships between countries and institutions are extensive. China and USA are the countries with the closest cooperation, and they also cooperate most extensively with other countries. From the cluster analysis of institutions, it can be seen that breast cancer and skin cancer are the cancers that are studied more collaboratively across institutions, and their research is mainly on the diagnosis, classification and detection of cancer. However, the cooperation relationships between authors are relatively lacking. We all known that the identity of authors includes doctors and scientists. Because of their different identities, the perspective of the same problem may also be different, and further exploring the collaboration between doctors and scientists may bring us more surprises. But unfortunately, the lack of cooperation between authors and corresponding data support, this study is very difficult and requires further efforts. In short, cooperation is one of the effective ways to achieve breakthroughs and win–win, although some achievements have been made in the application of DL in cancer, more extensive and in-depth cooperation is still needed.

Analysis of journals shows that the top ten journals cover medicine, computer science, and biomedicine, while medical journals are mainly concentrated in the fields of oncology and medical imaging. This gives researchers a preliminary impression of the application of DL in cancer: DL is a subfield of ML, the application of DL in cancer is an interdisciplinary field involving medicine and computer science. The application of DL in cancer builds on earlier research in mathematics, computer science, and molecular biology, utilizing complex algorithms such as CNNS and RNNS to play an important role in the diagnosis, prediction, prognosis assessment, discovery of new biomarkers of modern medicine. At the same time, analysis of journals provides researchers with follow-up research direction and the selection of journals. Researchers can determine their research priorities based on the thematic direction of high-impact journals and select appropriate journals for submission. In addition, the interdisciplinary landscape displayed in dual-map overlay of journals reveals major applications of DL in cancer: 1) Tumor detection and classification: using DL technology to detect and classify tumors early and improve diagnostic accuracy. 2) Medical imaging: using DL technology in medical imaging technology such as CT and MRI to achieve more accurately identify the lesion. 3) Cancer prognosis prediction: using DL technology to predict the progression and prognosis of cancer by analyzing clinical data and genetic information of patients. 4) Biomarker discovery: using DL technology to discover new cancer biomarkers and promote the development of precision medicine [33–36].

Co-citation of references is a reliable indicator to indicate the research basis, which can identify the landmark references and the evolution of research topics. In this study, the most cited reference is the “*ImageNet Classification with Deep Convolutional Neural Networks*” authored by *Krichevsky* [5]. In 2012, *Krichevsky* won the first place in the *ILSVRC-2012* competition, which marked the arrival of the era of DL and laid the foundation for the further application of DL. Further analysis of cluster analysis of co-cited references, which indicates the evolution of the application of DL in cancer. In #0 “mitosis detection” cluster, researchers proposed different DL models for mitotic detection of breast cancer. In 2016, *Albarqouni* proposed an additional crowdsourcing layer (*AggNet*) for mitosis of breast cancer histological images, the data aggregation is processed directly through the *AggNet* and as part of CNNs [37]. In 2018, *Li* proposed a multi-stage DL framework to accurately detect mitotic cells in pathological sections [38]. Next, as shown in #5 “breast cancer diagnosis” and #6 “breast cancer classification” clusters, more and more DL models were applied to the diagnosis and classification of the breast cancer. In 2019, *Ragab* proposed a new computer aided detection (CAD) systema for the classification of benign and malignant breast mass. The CAD system used two segmentation methods and achieved the highest area under the curve compared to the previous ones [39]. *Shen* also proposed a DL algorithm in 2019 for breast cancer diagnosis, which used a DL algorithm with an “end-to-end” training method, reducing the reliance on rarely available lesion annotations [40]. In 2020, *Liu* applied a DL method based on *Bilinear Convolutional Neural Networks* to fine-grained classification of breast cancer and achieved an accuracy rate of more than 95% [41]. At the same time, different DL algorithms are also used for the classification, prediction, detection, diagnosis of skin cancer and lung cancer [42–45]. Recently, DL algorithms have even been used to predict genetic mutations. In 2021, a multi-channel and multi-task DL model proposed by *Dong* was used to predict *EGFR* and *KRAS* mutations in non-small cell lung cancer, with a prediction accuracy of about 70% [46].

Keywords are important index to reflect the research topic. The frequency of keywords can reflect the current research status and hotspots, while the burst detection of keyword can reflect the evolution of research. In our study, by analyzing the burst detection of keywords, we notice that the earliest burst keyword is “convolutional neural network”. CNNs is a class of DL models specifically designed to process data with a grid structure, it is mainly composed of convolutional layer, activation function, pooling layer and fully connected layer. Because of its automatic feature extraction, parameter sharing and spatial invariance, CNNs is widely used in various image processing tasks and in DL [47, 48]. Other common used DL networks and reasoning models also include RNNs, Generative Adversarial Networks (GANs), Variational Autoencoders (VAEs) [49, 50]. Besides, other keywords are related to imaging, pathology, and specific cancer names. Therefore, we concluded that the application of DL in cancer is mainly related to imaging. Combined with radiomics and DL, DL models are used for cancer prediction, detection, classification, diagnosis, and prognosis [51]. For example, *Paul* used DL to predict the benign and malignant pulmonary nodules [52], *Heuvelmans* retrospectively verified the accuracy of DL in identify the benign and malignant pulmonary nodules, confirming that DL can be used as an effective tool for classifying and differentiating pulmonary malignant nodules [53]. In addition, analysis of keywords shows that the application of DL in breast cancer, skin cancer, and lung cancer has been relatively mature, while the application of oral cancer and thyroid cancer has been some study [54, 55], but it is still in its infancy. The burst detection of keywords can find the keywords with strongest citation bursts in a certain period of time, and indicate research hotspots and trends. The application of DL in cancer has experienced early research on DL models and algorithms. With the gradual maturity of various algorithms, DL technology has begun to be applied in practice, various DL algorithms have been widely used in the diagnosis, classification, and prediction of lung nodules and breast masses. In 2023, the main keywords with strongest citation bursts are “predictive models”, “resection”, and “protein”. This indicates that in addition to further expanding the application scope of and accuracy of DL models, protein structure prediction and genomics based on DL will be another research hotspots [56–58]. To sum up, based on our research, we summarized the application of DL in cancer as follows: first, to achieve early detection and diagnosis of cancer in combination with medical imaging technology; second, to perform prognostic analysis based on clinical data and genetic data; third, to improve the efficiency and accuracy of image processing by using automatic image segmentation and processing; fourth, to discover new cancer biomarkers through extensive biological data and promote the development of precision medicine. In the future, the application of DL in cancer is to integrate DL, protein prediction, genomics and cancer through interdisciplinary cooperation to further realize precision medicine [59].

However, this study still had some limitations. First, the search process may have led to the omission of some important articles. This omission could be attributed to two main reasons. Firstly, DL is a sub-branch of AI and ML, and some AI and ML articles may include studies related to DL. Since we aimed to analyze this small sub-branch of DL, some relevant articles may have been inadvertently excluded. Secondly, while the WoSCC database is a large and comprehensive citation database, it may still miss some important articles that only included in other databases. Second, the study included only English-language articles, but some important non-English literatures may have been overlooked. Finally, bibliometric analysis is a descriptive study that only analyzes the current state of research at a specific time. But medical science is constantly evolving, various new articles are published every day, so time constraints should be taken into account when analyzing. Therefore, it is important to acknowledge that new and constantly improving research is still needed in the future to compensate for the current limitations.

## Conclusion

In this study, we used bibliometric analysis to explore the research basis, research status, research hotspots and future research trends of DL in cancer. Overall, the application of DL in cancer is a highly promising research area that has attracted the interest of many researchers in recent years. Presently, DL has been widely used in the prediction, detection, classification, diagnosis and prognosis of breast cancer, lung cancer, skin cancer, while the application of oral cancer and thyroid cancer is still in its infancy. In the future, further expanding the application scope of DL and improving the accuracy of DL models will be hotspots. At the same time, integrating DL with protein prediction, genomics and cancer will be another future research trends. Continuous advancements in these areas will further enhance the capabilities and impact of DL in cancer research.

## Data Availability

The data for this study were obtained from the Web of Science database (http://webofknowledge.com).

## References

[1] McCarthy J, Minsky M, Rochester N (2006). A proposal for the dartmouth summer research project on artificial intelligence, august 31, 1955. AI Mag.

[2] Choi RY, Coyner AS, Kalpathy-Cramer J, Chiang MF, Campbell JP (2020). Introduction to machine learning, neural networks, and deep learning. Transl Vis Sci Technol.

[3] Sultan AS, Elgharib MA, Tavares T, Jessri M, Basile JR (2020). The use of artificial intelligence, machine learning and deep learning in oncologic histopathology. J Oral Pathol Med.

[4] Hinton GE, Osindero S, Teh YW (2006). A fast-learning algorithm for deep belief nets. Neural Comput.

[5] Krizhevsky A, Sutskever I, Hinton GE (2012). ImageNet classification with deep convolutional neural networks. Adv Neural Inf Process Syst.

[6] Alzubaidi L, Zhang J, Humaidi AJ (2021). Review of deep learning: concepts, CNN architectures, challenges, applications, future directions. J Big Data.

[7] Qin Z, Ye H, Li GY, Juang BH (2019). Deep learning in physical layer communications. IEEE Wirel Commun.

[8] Wang TQ, Wen CK, Wang HQ, Gao FF, Jiang T, Jin S (2017). Deep learning for wireless physical layer: Opportunities and challenges. China Communications.

[9] Michelucci, U. Convolutional and Recurrent Neural Networks. Applied Deep Learning. Apress, Berkeley, CA. 2018. 10.1007/978-1-4842-3790-8_8.

[10] Voulodimos A, Doulamis N, Doulamis A, Protopapadakis E (2018). Deep learning for computer vision: a brief review. Comput Intell Neurosci..

[11] Kłosowski P. Deep learning for natural language processing and language modelling. In: Signal Processing: Algorithms, Architectures, Arrangements, and Applications (SPA). Poznan; 2018. p. 223–228. 10.23919/SPA.2018.8563389.

[12] Cao C, Liu F, Tan H (2018). Deep learning and its applications in biomedicine. Genomics Proteomics Bioinformatics.

[13] Chan HP, Samala RK, Hadjiiski LM, Zhou C (2020). Deep learning in medical image analysis. Adv Exp Med Biol.

[14] Zou J, Huss M, Abid A, Mohammadi P, Torkamani A, Telenti A (2019). A primer on deep learning in genomics. Nat Genet.

[15] Wang Z, Majewicz FA (2018). Deep learning with convolutional neural network for objective skill evaluation in robot-assisted surgery. Int J Comput Assist Radiol Surg.

[16] Balkenende L, Teuwen J, Mann RM (2022). Application of deep learning in breast cancer imaging. Semin Nucl Med.

[17] Dildar M, Akram S, Irfan M (2021). Skin cancer detection: a review using deep learning techniques. Int J Environ Res Public Health.

[18] Wu Y, Chen B, Zeng A, Pan D, Wang R, Zhao S (2022). Skin cancer classification with deep learning: a systematic review. Front Oncol.

[19] Wang L (2022). Deep learning techniques to diagnose lung cancer. Cancers (Basel).

[20] Gupta A, Parveen A, Kumar A, Yadav P (2022). Advancement in deep learning methods for diagnosis and prognosis of cervical cancer. Curr Genomics.

[21] Sahiner B, Pezeshk A, Hadjiiski LM (2019). Deep learning in medical imaging and radiation therapy. Med Phys.

[22] Summers RM (2019). Are we at a crossroads or a plateau? Radiomics and machine learning in abdominal oncology imaging. Abdom Radiol (NY).

[23] Khairi SSM, Bakar MAA, Alias MA (2021). Deep learning on histopathology images for breast cancer classification: A bibliometric analysis. Healthcare (Basel).

[24] Zhong R, Gao T, Li J (2024). The global research of artificial intelligence in lung cancer: a 20-year bibliometric analysis. Front Oncol.

[25] Zhang G, Song J, Feng Z (2023). Artificial intelligence applicated in gastric cancer: A bibliometric and visual analysis via CiteSpace. Front Oncol.

[26] Esteva A, Kuprel B, Novoa RA (2017). Dermatologist-level classification of skin cancer with deep neural networks [published correction appears in Nature. 2017 Jun 28;546(7660):686]. Nature.

[27] Brinker TJ, Hekler A, Hauschild A (2019). Comparing artificial intelligence algorithms to 157 German dermatologists: the melanoma classification benchmark. Eur J Cancer.

[28] Tran KA, Kondrashova O, Bradley A, Williams ED, Pearson JV, Waddell N (2021). Deep learning in cancer diagnosis, prognosis and treatment selection. Genome Med..

[29] Sharma J (2016). Digital India and its Impact on the Society. International Journal of Research in Humanities and Soc. Sciences.

[30] Jindal N, Thakur K, Sharma T (2019). Digital India: Challenges, Solutions and Its Impact on Society. International Journal of Environment, Ecology, Family and Urban Studies (IJEEFUS).

[31] Chatterjee S (2020). AI strategy of India: policy framework, adoption challenges and actions for government. Transforming Government: People, Process and Policy.

[32] Rodriguez RV, Sinha S, Tripathi S (2020). Impact of Artificial Intelligence on the health protection scheme in India. Public Administration and Policy.

[33] Nazir M, Shakil S, Khurshid K (2021). Role of deep learning in brain tumor detection and classification (2015 to 2020): A review. Comput Med Imaging Graph.

[34] Currie G, Hawk KE, Rohren E, Vial A, Klein R (2019). Machine learning and deep learning in medical imaging: intelligent imaging. J Med Imaging Radiat Sci.

[35] Poirion OB, Jing Z, Chaudhary K, Huang S, Garmire LX (2021). DeepProg: an ensemble of deep-learning and machine-learning models for prognosis prediction using multi-omics data. Genome Med.

[36] Mathema VB, Sen P, Lamichhane S, Orešič M, Khoomrung S (2023). Deep learning facilitates multi-data type analysis and predictive biomarker discovery in cancer precision medicine. Comput Struct Biotechnol J.

[37] Albarqouni S, Baur C, Achilles F, Belagiannis V, Demirci S, Navab N (2016). Aggnet: deep learning from crowds for mitosis detection in breast cancer histology images. IEEE Trans Med Imaging.

[38] Li C, Wang X, Liu W, Latecki LJ (2018). DeepMitosis: Mitosis detection via deep detection, verification and segmentation networks. Med Image Anal.

[39] Ragab DA, Sharkas M, Marshall S, Ren J (2019). Breast cancer detection using deep convolutional neural networks and support vector machines. PeerJ.

[40] Shen L, Margolies LR, Rothstein JH, Fluder E, McBride R, Sieh W (2019). Deep learning to improve breast cancer detection on screening mammography. Sci Rep.

[41] Liu W, Juhas M, Zhang Y (2020). Fine-grained breast cancer classification with bilinear convolutional neural networks (BCNNs). Front Genet..

[42] Pacheco AGC, Krohling RA (2021). An attention-based mechanism to combine images and metadata in deep learning models applied to skin cancer classification. IEEE J Biomed Health Inform.

[43] Huang HW, Hsu BW, Lee CH, Tseng VS (2021). Development of a light-weight deep learning model for cloud applications and remote diagnosis of skin cancers. J Dermatol.

[44] Ardila D, Kiraly AP, Bharadwaj S, Choi B, Reicher JJ, Peng L, Tse D, Etemadi M, Ye W, Corrado G, Naidich DP (2019). End-to-end lung cancer screening with three-dimensional deep learning on low-dose chest computed tomography. Nat Med.

[45] Chen W, Hou X, Hu Y, Huang G, Ye X, Nie S (2021). A deep learning- and CT image-based prognostic model for the prediction of survival in non-small cell lung cancer. Med Phys.

[46] Dong Y, Hou L, Yang W (2021). Multi-channel multi-task deep learning for predicting EGFR and KRAS mutations of non-small cell lung cancer on CT images. Quant Imaging Med Surg.

[47] Liimatainen K, Huttunen R, Latonen L, Ruusuvuori P (2021). Convolutional neural network-based artificial intelligence for classification of protein localization patterns. Biomolecules.

[48] Liu J, Fu F (2023). Convolutional neural network model by deep learning and teaching robot in keyboard musical instrument teaching. PLoS One.

[49] Yasaka K, Akai H, Kunimatsu A, Kiryu S, Abe O (2018). Deep learning with convolutional neural network in radiology. Jpn J Radiol.

[50] Xiao Y, Wu J, Lin Z (2021). Cancer diagnosis using generative adversarial networks based on deep learning from imbalanced data. Comput Biol Med.

[51] Avanzo M, Wei L, Stancanello J (2020). Machine and deep learning methods for radiomics. Med Phys.

[52] Paul R, Hawkins SH, Schabath MB, Gillies RJ, Hall LO, Goldgof DB (2018). Predicting malignant nodules by fusing deep features with classical radiomics features. J Med Imaging (Bellingham).

[53] Heuvelmans MA, van Ooijen PMA, Ather S (2021). Lung cancer prediction by Deep Learning to identify benign lung nodules. Lung Cancer.

[54] Kim DW, Lee S, Kwon S, Nam W, Cha IH, Kim HJ (2019). Deep learning-based survival prediction of oral cancer patients. Sci Rep.

[55] Peng S, Liu Y, Lv W, Liu L, Zhou Q, Yang H, Ren J, Liu G, Wang X, Zhang X, Du Q (2021). Deep learning-based artificial intelligence model to assist thyroid nodule diagnosis and management: a multicentre diagnostic study. Lancet Digit Health.

[56] Pakhrin SC, Shrestha B, Adhikari B, Kc DB (2021). Deep learning-based advances in protein structure prediction. Int J Mol Sci..

[57] Torrisi M, Pollastri G, Le Q (2020). Deep learning methods in protein structure prediction. Comput Struct Biotechnol J.

[58] Eraslan G, Avsec Ž, Gagneur J, Theis FJ (2019). Deep learning: new computational modelling techniques for genomics. Nat Rev Genet.

[59] MacEachern SJ, Forkert ND (2021). Machine learning for precision medicine. Genome..

